# Nutritional Status of Maintenance Dialysis Patients: Low Lean Body Mass Index and Obesity Are Common, Protein-Energy Wasting Is Uncommon

**DOI:** 10.1371/journal.pone.0150012

**Published:** 2016-02-26

**Authors:** Mette Koefoed, Charles Boy Kromann, Sophie Ryberg Juliussen, Danni Hvidtfeldt, Bo Ekelund, Niels Erik Frandsen, Peter Marckmann

**Affiliations:** 1 Department of Internal Medicine, section of Nephrology, Holbæk Hospital, Health Sciences faculty, University of Copenhagen, Holbaek, Denmark; 2 Department of Dermatology, Roskilde Hospital, Health Sciences faculty, University of Copenhagen, Roskilde, Denmark; 3 Department of Nutrition, Exercise and Sports, University of Copenhagen, Copenhagen N, Denmark; 4 Department of Internal Medicine, section of Nephrology, Roskilde Hospital, Health Sciences faculty, University of Copenhagen, Roskilde, Denmark; University of Palermo, ITALY

## Abstract

**Background and Aims:**

Maintenance dialysis patients are at increased risk of abnormal nutritional status due to numerous causative factors, both nutritional and non-nutritional. The present study assessed the current prevalence of protein-energy wasting, low lean body mass index and obesity in maintenance dialysis patients, and compared different methods of nutritional assessment.

**Methods:**

In a cross-sectional study conducted in 2014 at Roskilde Hospital, Denmark, we performed anthropometry (body weight, skinfolds, mid-arm, waist, and hip circumferences), and determined plasma albumin and normalized protein catabolic rate in order to assess the prevalence of protein-energy wasting, low lean body mass index and obesity in these patients.

**Results:**

Seventy-nine eligible maintenance dialysis patients participated. The prevalence of protein-energy wasted patients was 4% (95% CI: 2–12) as assessed by the coexistence of low lean body mass index and low fat mass index. Low lean body mass index was seen in 32% (95% CI: 22–44). Obesity prevalence as assessed from fat mass index was 43% (95% CI: 32–55). Coexistence of low lean body mass index and obesity was seen in 10% (95% CI: 5–19). The prevalence of protein-energy wasting and obesity varied considerably, depending on nutritional assessment methodology.

**Conclusions:**

Our data indicate that protein-energy wasting is uncommon, whereas low lean body mass index and obesity are frequent conditions among patients in maintenance dialysis. A focus on how to increase and preserve lean body mass in dialysis patients is suggested in the future. In order to clearly distinguish between shortage, sufficiency and abundance of protein and/or fat deposits in maintenance dialysis patients, we suggest the simple measurements of lean body mass index and fat mass index.

## Introduction

Dialysis patients are at high risk of abnormalities in their nutritional status due to uremic anorexia, dietary limitations, physical inactivity, chronic inflammation, co-morbidities, and metabolic derangements[1]. Many clinical studies have demonstrated the frequent presence of undernourished and protein-energy wasted (PEW) dialysis patients[2,3]. The reported prevalence varies from 20 to 75%, in part depending on characteristics of the study population, but also depending on differences in methodology and diagnostic criteria. Recent studies have shown that obesity also is a frequent condition in these patients [4,5].

Only a few previous studies have simultaneously assessed both PEW and obesity prevalence in maintenance dialysis patients[6,7]. These studies reported a confusing overlap between patients with PEW (i.e. protein and energy shortage) and those with obesity (i.e. energy abundance), indicating a problem with nutritional assessment methodologies. Information about whether patients are in shortage or abundance of protein and/or energy deposits is necessary for the future nutritional guidance given by nephrologists and dieticians to dialysis patients.

In the present study, we therefore aimed at estimating PEW and obesity prevalence with a method that could clearly distinguish between patients with shortage, sufficiency and abundance of protein and energy deposits. This method is based on simple measurements of lean body mass index and fat mass index, which have been shown to provide valuable information about body composition in healthy adults [8]. As an integral part of the study, we also assessed PEW and obesity prevalence with alternative, established methods in order to elucidate any problems associated with these commonly used methods.

## Materials and Methods

### Design

In a cross-sectional study, we investigated the nutritional status and body composition of hemodialysis (HD) and peritoneal dialysis (PD) patients attending the dialysis centre at Roskilde Hospital, Denmark, in February to June 2014.

### Participants

All PD, HD and home-HD patients (n = 105) were invited to participate. Exclusion criteria were: dialysis vintage less than 3 months, fever, current antibiotic treatment, major surgery within two weeks, disseminated cancer, age below 18 years, psychosis, pregnancy, language barriers and physical or mental disability making participation unfeasible. Patient data (age, gender, dialysis vintage, primary kidney disease and co-morbidity) were taken from patient records.

### Ethics

All participants gave their written and informed consent. The local research ethics committee (RVK Sjælland) approved the study protocol (file no. 40480), and all procedures were in accordance with Helsinki Declaration of 1975. The study was registered at ClinicalTrial.gov (ID: NCT02320552).

### Anthropometric measurements

Two trained research assistants (authors SRJ and DH) performed all anthropometric measurements immediately after a dialysis session (HD), or after the monthly control in the outpatient clinic (PD, home-HD). All participants were considered normohydrated. If patients were obviously overhydrated anthropometric measurements were postponed.

Height and body weight (BW) were measured in light clothing using standard instruments. For PD patients, weighing was preferably done with empty abdominal cavity, but for eight patients who refused to empty their abdominal cavity, we defined BW as measured weight with dialysate minus the volume of dialysate that was last instilled into the abdomen. For two patients with lower leg amputations, BW was assessed as actual weight plus 6,3% of actual weight per amputated leg[9].

Skinfolds were measured with a Harpenden caliper. Mid-arm, waist and hip circumferences were assessed with a non-stretchable fiberglass insertion tape (seca 201, Seca, Hamburg, Germany). Fat mass (FM) was assessed according to Durnin and Womersley based on four skinfold thicknesses (biceps, triceps, supscapular and suprailiac)[10]. Lean body mass (LBM) was calculated from the equation LBM = BW—FM. Fat mass index (FMI = FM/h^2^) and lean body mass index (LBMI = LBM/h^2^) were then determined. Mid-arm circumference (MAC) was measured midway between acromion and olecranon. Triceps skinfold and MAC were used to calculate corrected mid-arm muscle area (cMAMA)[11].

All skinfold and circumference measurements were done in duplicate on the non-access side of the body for HD patients, and on the right side for PD patients. If two measurements differed more than four mm, two additional measurements were performed, and the mean of all four was used for the analyses[11].

Waist circumference (WC), hip circumference, and waist-hip ratio (WHR) was measured as recommended by WHO[12]. Four patients unable to stand had their circumferences measured in supine position[13]. Measurements were not performed in 8 PD patients who refused to empty their abdominal cavity. All measurements were done in duplicate. If two measurements differed more than one cm, two additional measurements were undertaken, and the mean of all four used in the analyses.

### Anthropometric measurement inter-observer reliability

To ensure agreement between the two observers (authors SRJ and DH), both observers measured ten healthy subjects in order to perform a two-way random, single measure Intraclass Correlation Coefficient (ICC) analysis. ICC was 0.79 (p = 0.002) for sum of 4 skinfolds indicating good agreement. For MAC and WHR, the ICC was 0.95 (p<0.001) and 0.97 (p<0.001) indicating almost perfect agreement [14,15].

### Blood sampling and biochemical measurements

For assessment of plasma albumin, blood samples were immediately analyzed with bromocresol purple at the laboratory at Roskilde Sygehus, Denmark. Kt/V was measured as described by Gotch [16]. For HD patients, normalized protein catabolic rate (nPCR) was determined by measuring the interdialytic rise of blood urea nitrogen after a midweek dialysis session [17]. For patients with residual urine output > 300 ml/day (n = 24), urinary nitrogen excretion was taken into account. To account for day-to-day variations we calculated a mean of three nPCR measurements made over a two months period in HD patients. However, only one urine sample was collected for each patient. For PD-patients, nPCR was measured using Bergstrom's equation [18]. Only one nPCR measurement was done for each PD patient.

### PEW and low LBMI

According to an International Society of Renal Nutrition and Metabolism (ISRNM) expert panel paper, PEW is a "state of decreased body stores of protein and energy fuels (that is, body protein and fat masses)"[19]. We therefore classified our patients as having PEW, if they had both LBMI (a measure of body stores of protein) and FMI (a measure of body stores of energy fuels) below the 10th percentile of a reference population[20]. In this paper, the term PEW_LBMI, FMI_ is used for this condition.

We also assessed PEW prevalence with the methodology recommended by ISRNM and denoted it PEW_ISRNM_ [19]. According to ISRNM, PEW_ISRNM_ may be diagnosed if a patient has at least one abnormal value in at least three of four categories of nutritional variables. We selected the following ISRNM-specified PEW-indicators from each of the four defined categories: P-albumin < 38 g/L, BMI < 23 kg/m^2^, cMAMA < 90% of median of reference population, and dietary protein intake < 0.8 g/kg/d. With respect to cMAMA, we used reference data published by Frisancho, which to our knowledge is the only relevant published dataset[21]. This dataset only covers individuals aged 18–75 years. For patients older than 75 years, reference values were extrapolated based on an expected 5% decline per 5 years for men, and a 3% decline per 5 years for women as found in a recent Irish study [22]. We used nPCR as a surrogate for dietary protein intake, which is acceptable in clinically stable patients.

Low LBMI was defined as a LBMI < 10th percentile of a reference population[20].

### Obesity

We used three different definitions of obesity: the WHO definition (BMI > 30 kg/m^2^), a definition based on fat mass percentage (FM%) (men (M) > 25%, women (W) >35%[23]), and one based on FMI (> 90 percentile of a age-stratified reference population). Presence of abdominal obesity was defined from either WC (M > 102 cm, F > 88 cm) or WHR (M > 0.90, F > 0.85)[12]. The term obese sarcopenia has previously been used for a condition with low muscle mass despite of fat accumulation[6,7,24]. We defined obese sarcopenia as concomitant FMI > 90^th^ percentile and LBMI < 10^th^ percentile of reference population.

### Statistics

Shapiro-Wilks’ test was used to examine normal distribution. If data followed normal distribution, mean ± SD was reported and unpaired Student’s t-test was applied to compare means. If data did not follow normality, median (range) was reported and Mann-Whitney's U statistics was applied to compare groups. When comparing rates, Fisher’s exact test or chi-squared analysis were applied as appropriate. Confidence intervals [95% CI] of prevalences were calculated using Wilson’s score interval. A p ≤ 0.05 was considered significant.

Unless otherwise noted, all statistic calculations were performed using IBM SPSS Statistics version 22 for Mac OS X (IBM Corp. Worldwide).

## Results

### Participant characteristics

From our total patient population (n = 105), 10 HD and three PD patients had to be excluded (5 due to mental or physical disability, 5 due to disseminated cancer, two due to language barriers, and one due to acute infection). Another 9 patients declined the invitation to participate. Among the remaining 83 patients included in the study, three patients died, and one had a renal transplant before their examination.

The median age of the 79 study participants was 67.9 (range: 22–89) years, and their median dialysis vintage was 21.9 (range: 3–117) months (Table 1). There was no difference between participants and non-participants regarding age, dialysis vintage, p-albumin or BMI. A minority of patients (n = 11) declined to accept one or more of the planned measurements during the study, so complete datasets were only available for 68 patients.

**Table 1:** Characteristics of maintenance dialysis patients at Roskilde Hospital, Denmark (n = 79).

	Parameter	HD n = 44	PD n = 35	P^1^
**Basic variables**				
	Men (%)	75	77	NS
	Age (years)	64.6 ±12.5	66.5 ±11.5	NS
	Dialysis vintage (months)	26.2 (8.9–114.3)	19.3 (3.2–117.2)	< 0.05
	24-hour urine (L)	0.39 (0–2.83)	1.5 (0–4.0)	<0.001
	CVD (%)	11	26	NS
	DM (%)	27	9	NS
**Renal ethiologies**				
	Glomerulonephritis (%)	16	29	22
	Diabetic nephropathy (%)	23	3	14
	Others and unknown (%)	61	68	64
**Biochemical variables**				
	Albumin (g/L)	33 ±5	31±5	NS
	nPCR (g/kg/d)^2^	0.94 ±0.19	0.85 ± 0.24	NS
**Anthropometric variables**				
	BMI (kg/m^2^)	25.2 ±5.1	27.3 ±3.9	<0.05
	cMAMA (M) (cm^2^)	42.2 (16.2–70.3)	41.5 (28.5–62.2)	NS
	cMAMA (W) (cm^2^)	36.5 (21.4–59.7)	34.3 (26.6–41.2)	NS
	WC (M) (cm)^3^	100 ±14	110 ±12	<0.05
	WC (W) (cm)	95 ±12	98 ±8	NS
	WHR (M)	1.01 (0.92–1.21)	1.07 (0.96–1.19)	NS
	WHR (W)	0.97 (0.79–1.03)	0.93 (0.90–0.99)	NS
	FM% (M) (%)^4^	27.8 ±7.5	32.1 ±7.0	<0.05
	FM% (W) (%)	35.6 ±9.3	39.3 ±6.7	NS
	FM (M) (kg)	22.8 ±10.0	28.5 ±10.3	<0.05
	FM (W) (kg)	26.3 ±14.3	28.1 ±7.8	NS
	LBM (M) (kg)	55.7 ±9.0	58.1 ±8.1	NS
	LBM (W) (kg)	43.1 ±6.3	42.6 ±4.5	NS
	FMI (M) (kg/m^2^)	7.2 ±3.0	9.1 ± 3.0	<0.05
	FMI (W) (kg/m^2^)	9.5 ±4.4	10.6 ±3.3	NS
	LBMI (M) (kg/m^2^)	17.8 ±2.4	18.5 ±1.5	NS
	LBMI (W) (kg/m^2^)	16.0 ±1.0	15.9 ±1.8	NS

Figures are mean ± SD, median (range) or percentage.

cMAMA: corrected mid-arm muscle area; CVD: cardiovascular disease; DM: diabetes mellitus; FM: fat mass; FM%: fat percentage; FMI: fat mass index; HD: hemodialysis patients; LBM: lean body mass; LBMI: lean body mass index; M: men; nPCR: normalized protein catabolic rate; NS: not significant; PD: peritoneal dialysis patients; WC: waist circumference; WHR: waist hip ratio; W: women.

^1^p for difference between HD and PD patients. Calculated using Student’s t-test or Mann-Whitney's U when appropriate

^2^nPCR obtained from 43 HD and 34 PD patients

^3^WC and WHR measured in 26 HD men, 8 HD women, 21 PD men, and 5 PD women

^4^Body composition (FM, LBM and derivates thereof) assessed in 26 HD men, 8 HD women, 26 PD men, and 8 PD women

HD patients had significantly longer dialysis vintage and a lower 24-hour urine output than PD patients (Table 1). In addition, HD patients had significantly lower BMI. HD men had significantly lower WC, FM%, FM and FMI than PD men. Among women, similar insignificant trends were observed.

### Prevalence of PEW

**Fig 1:**
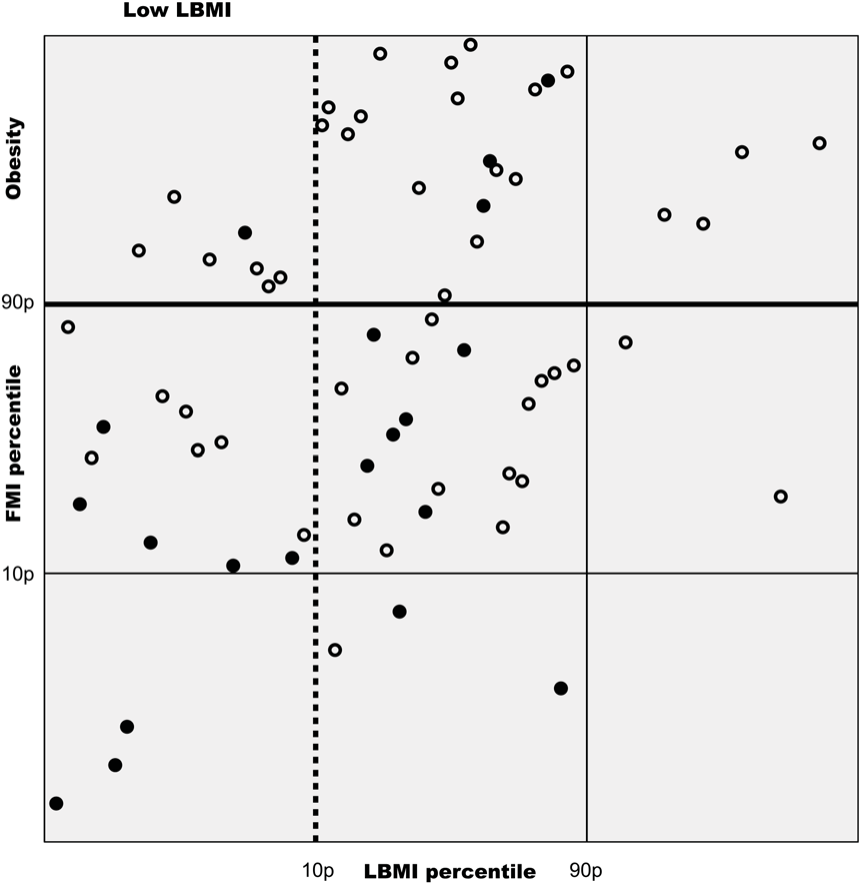
Non-linear plot of fat mass index (FMI) versus lean body mass index (LBMI) of maintenance dialysis patients (n = 68). Horizontal and vertical lines indicate 10th (10p) and 90th percentile (90p) of healthy reference population. Filled circles are patients classified as protein-energy wasted according to ISRNM criteria. The horizontal bold line separates obese individuals from the non-obese and the vertical dotted line separates individuals with low LBMI from the individuals with normal or high LBMI.

The prevalence of PEW was 4% (95% CI: 2–12) according to LBMI and FMI measurements (PEW_LBMI, FMI_). In contrast, 29% (95% CI: 20–41) of the participants had PEW according to the ISRNM methodology (PEW_ISRNM_) (p < 0.01 for difference between methods) (Table 2). The strong disagreement between the two methods is illustrated in Fig 1. Only a small proportion (n = 3, 15%) of the 20 patients with PEW_ISRNM_ did in fact have both low body stores of protein and energy fuels as assessed from LBMI and FMI.

**Table 2:** Prevalence (% [95% confidence interval]) of different nutritional states in maintenance dialysis patients at Roskilde Hospital, Denmark (n = 68)).

		HD	PD	HD+PD
		n = 34	n = 34	n = 68
**PEW**				
	PEW_LBMI, FMI_	9 [3–23]	0 [0–10]	4 [2–12]
	PEW_ISRNM_	35 [22–52]	24 [12–40]	29 [20–41]
**Obesity**				
	Obesity_BMI_	18 [8–34]	24 [12–40]	21 [13–32]
	Obesity_FMI_	32 [19–49]	53 [37–69]	43 [32–55]
	Obesity_FM%_	65 [48–79]	82 [67–92]	74 [62–83]
**Underweight**		6 [2–20]	0 [0–10]	3 [1–10]
**Low LBMI**		41 [26–58]	24 [12–40]	32 [22–44]
**Obese sarcopenia**_**LBMI, FMI**_		9 [3–23]	12 [5–27]	10 [5–19]

Abbreviations: FM%: body fat percent; FMI: fat mass index; HD: hemodialysis patients; LBMI: lean body mass index; Low LBMI: LBMI < 10^th^ percentile of reference population; Obese sarcopenia_LBMI,FMI_: LBMI < 10^th^ percentile and FMI > 90^th^ percentile of reference population; Obesity_BMI:_ BMI > 30 kg/m^2^; Obesity_FM%_: FM% > 25 (men) or > 35 (women); Obesity_FMI_: fat mass index > 90th percentile of reference population; PD: peritoneal dialysis patients; PEW: protein-energy wasting; PEW_ISRNM_: PEW according to criteria defined by International Society of Renal Nutrition and Metabolism; PEW_LBMI, FMI_: LBMI < 10^th^ percentile and FMI < 10^th^ percentile of reference population; Underweight: BMI < 18,5kg/m^2^.

Patients were divided into low (< 10th percentile), normal (10–90th percentile), and high (> 90th percentile) values for each variable. Within each of the 3 subgroups, patients were ranked according to their absolute LBMI and FMI.

### Prevalence of low LBMI

Low LBMI was found in 32% (95% CI: 22–44) (n = 22). The combination of low LBMI and high FMI—the so-called obese sarcopenic phenotype—was seen in 10% (95% CI: 5–19, n = 7)(Table 2).

### Prevalence of obesity

The prevalence of obesity varied largely and statistically significantly depending on diagnostic criterion (Table 2). Abdominal obesity was seen in 58% (95% CI: 46–70) according to WC, and in 98% (95% CI: 91–100) according to WHR (p < 0.01 for difference between methods). Based on WC assessments, abdominal obesity was significantly more frequent among PD (81%) than HD patients (41%)(p = 0.002). Only two patients (3%, 95% CI: 1–10) were underweight as defined by WHO (BMI < 18.5 kg/m^2^). Underweight was only seen in HD patients.

### PEW versus obesity

Patients diagnosed with PEW_ISRNM_ were frequently also classified as obese. As seen from Fig 1, 20% (n = 4) of 20 individuals with PEW_ISRNM_ were obese according to FMI values. Regarding PEW_LBMI,FMI_, coexistence with obesity was not seen.

## Discussion

Our study suggests that PEW assessed from LBMI and FMI is uncommon among maintenance dialysis patients, whilst a large proportion of the study population have a low lean body mass index and/or are obese. Also, we found that different nutritional assessment methods lead to very different estimates of PEW and obesity prevalence. In the following sections, we discuss the validity of different methodologies.

According to ISRNM methodology, a considerable proportion (29%) of our study participants had PEW. Similar figures (15–50%) for PEW prevalence were reported from other recent studies using either ISRNM methodology[25] or the method of Subjective Global Assessment (SGA) [26–28]. However, the question is whether these estimates are valid. As previously noted, ISRNM defines PEW as a”state of decreased body stores of protein and energy fuels (that is, body protein and fat masses)”[19].

Our data demonstrated that among patients with PEW_ISRNM_, only a small proportion did in fact have both low body stores of protein and energy fuels as assessed from LBMI and FMI (Fig 1). Furthermore, a significant proportion of patients with PEW_ISRNM_ was obese, i.e. had large fat deposits. These observations indicate that ISRNM diagnostic criteria do not classify patients accurately according to ISRNM's own definition of PEW. The SGA methodology has also been criticized for misclassifying patients with respect to nutritional status[29].

In our opinion, the simple assessment of LBMI and FMI seems a better, easily understandable, and more reliable basis for the identification of patients with decreased body stores of protein and energy fuels. Based on LBMI and FMI, we suggest that PEW is unusual among Danish maintenance dialysis patients.

The prevalence of obesity also varied extensively depending on diagnostic criteria as previously reported [30,31]. Highest prevalence was seen if obesity was defined from FM%, followed by the FMI-based definition, whereas the BMI-based definition gave the lowest obesity prevalence (Table 2). As discussed by others, BMI is not an optimal measure of obesity, in particular not in groups of patients with extraordinary low or high lean body mass [32]. BMI is the sum of LBMI and FMI, and BMI therefore will tend to underestimate the prevalence of obesity in patients with low LBMI, such as patients with severe, chronic disease, including renal failure. The same limitations apply to FM%, which will tend to overestimate obesity in patients with poor lean body mass status. FMI is a favourable alternative for assessing obesity, because it is uninfluenced by lean body mass. According to FMI measurements, obesity was present in 43% (95% CI: 32–55) of our patients. A small minority of 9% (95% CI: 2–16) had FMI below the 10th percentile of a reference population indicating decreased body stores of energy fuels.

Parallel to the arguments for FMI, LBMI may be considered the best indicator of lean body mass status in patients, in particular those with unusual body composition. In our study, 32% of dialysis patients had low LBMI. The frequent presence of low LBMI might be explained by insufficient protein intake, increased protein catabolism, abnormally poor protein anabolism, or any combination of these. Our nPCR assessments showed that patients with low LBMI had a protein intake similar to that of patients with normal or high LBMI (data not shown) excluding insufficient dietary protein intake as the primary factor leading to low LBMI. In our population, decreased LBMI thus seems to stem from increased protein catabolism and/or abnormally poor protein anabolic activity. The presence of sarcopenic obesity in 10% of our patients underlines that lean body mass accretion and preservation may be poor, even when energy requirements are fully covered, indicating that an increase in physical activity or other anabolic strategies might be pivotal for the improvement of the nutritional status of many dialysis patients[1,33].

Some weaknesses and limitations of our study should be noted. The study was conducted at a single dialysis centre. Thus, our results may not be representative for dialysis populations from other centres. However, we have no indications from the national database of all Danish patients with renal failure to indicate that our patients should be different from other Danish patients[34].

We relied on skinfold measurements for the assessment of body fat. We would have preferred dual-energy X-ray absorptiometry (DEXA) or air displacement plethysmography for body composition assessments, but such gold standard methods were not available for the present study. However, others have demonstrated that skinfold-based estimates of body fat agree well with DEXA and ADP in HD patients[30,35,36]. For the classification of FMI and LBMI values, we used reference values derived from bioelectrical impedance analysis (BIA) measurements of a healthy Caucasian population[20]. In healthy subjects, BIA-based body composition assessments correspond well with a gold standard like DEXA [20]. In chronic kidney disease patients, skinfolds have better agreement with DEXA than BIA[36]. Accordingly, we found it acceptable to use BIA-based values from a healthy population as reference for our skinfold-based assessments.

For the measurement of plasma albumin, our lab used bromocresol purple (BCP) instead of bromocresol green (BCG) that is recommended by ISRNM. Parikh et al showed that BCP underestimates plasma albumin compared to BCG in both PD and HD patients, and suggested the use of a conversion formula from BCP to BCG [37]. When this formula was applied to our data, average albumin increased from 32.1 to 36.8 g/L and led to a change in PEW_ISRNM_ classification of only two patients. Thus, results would have remained largely unchanged and conclusions likewise.

ISRNM recommends that a least one abnormal value, but eventually any abnormal value of up to four markers in each of four nutritional marker categories may be included for diagnosing PEW [19]. We decided to measure only one variable from each category. If we had included more than one variable from each category, the PEW_ISRNM_ prevalence estimate would have been even higher as demonstrated by Gracia-Iguacel et al. They found that the prevalence of PEW_ISRNM_ increased from 40.5% to 47.3% by just adding one nutritional marker in one category [25]. ISRNM also suggests that each nutritional marker should optimally be confirmed on at least three occasions, preferably 2–4 weeks apart[19]. However, this is not a requirement, and we only measured each marker once, which is in line with what was practiced in a previous publication[25].

## Conclusion

Our study demonstrated that only 4% of our maintenance dialysis patients had PEW, whereas one third had low lean body mass index and almost half of the patients were obese. Our observations show that estimates of the prevalence of PEW and obesity vary strongly with nutritional assessment methodology, and that methods therefore should be selected with care. Finally, our findings indicate that the nutritional focus of nephrologists treating maintenance dialysis patients should be turned towards methods to increase and preserve lean body mass.
